# Bond-length distributions in ionically bonded materials with decomposition by coordination environment

**DOI:** 10.1107/S1600576722006884

**Published:** 2022-09-06

**Authors:** Motonari Sawada, Ryoga Iwamoto, Takao Kotani, Hirofumi Sakakibara

**Affiliations:** a Advanced Mechanical and Electronic System Research Center, Department of Engineering, Tottori University, Tottori, Japan; b Center for Spintronics Research Network (CSRN), Osaka University, Osaka, Japan; Instituto Andaluz de Ciencias de la Tierra, Granada, Spain

**Keywords:** bond-length distributions, ionic radii, Crystallography Open Database, *ChemEnv*, statistics

## Abstract

The distributions are analysed of the bond lengths between cations and anions in nitrides, oxides and fluorides for which structural data are available in the Crystallography Open Database (https://www.crystallography.net/cod/). The distributions are decomposed according to analysis of the coordination environments of the cations, which are offered in the tool *ChemEnv* [Waroquiers, George, Horton, Schenk, Persson, Rignanese, Gonze & Hautier (2020). *Acta Cryst.* B**76**, 683–695]. The distributions show how well the concept of ionic radius works. Chemical trends can be observed from the obtained distributions. For example, the bond-length distribution for Sc—O is similar to that for Zr—O. The distributions for Mo—O and V—O show noteworthy similarity. Resources for reproducing all of the results are available at https://github.com/tkotani/CEBond, with additional results for nitrides and fluorides.

## Abstract 2

To aid understanding of the idea of the ionic radius, a statistical analysis of the bond-length distributions for real crystal structures in the Crystallography Open Database is presented. A package to reproduce the results is freely available from https://github.com/tkotani/CEBond.

## 1. Introduction

The bond lengths between cations and anions in ionic crystals are some of the most important parameters to understand crystal structure. In this study, we analyse the bond-length distributions in nitrides, oxides and fluorides whose structural data are taken from the Crystallography Open Database (COD; https://www.crystallography.net/cod/; Vaitkus *et al.*, 2021 ▸; Quirós *et al.*, 2018 ▸; Merkys *et al.*, 2016 ▸; Gražulis *et al.*, 2009 ▸, 2012 ▸).

First of all, we note that the concept of atomic bond is a theoretical idea for us to understand a crystal structure. The atomic bonds in ionic materials can be uniquely defined only when we specify a theoretical model. As we show later, we use the software tool *ChemEnv* (Waroquiers *et al.*, 2017 ▸, 2020 ▸) as a method of determining atomic bonds.

In relation to the bond length, let us recall the idea of ionic radius. This was originally developed by Pauling and Goldschmidt (West, 2014 ▸; Pauling, 1960 ▸), and later Shannon and Prewitt statistically examined the radii (Shannon & Prewitt, 1969 ▸; Shannon, 1976 ▸). As the conclusion of this work, Shannon (1976 ▸) gave Table I (a copy of which is available at http://abulafia.mt.ic.ac.uk/shannon/radius.php), which gives two types of radius, the effective ionic radius (IR) and the crystal radius (CR). They determined the IR and CR by analysing the data for interatomic distances available at that time. The relation between IR and CR is simple: CR = IR + 0.14 Å for cations and CR = IR − 0.14 Å for anions. Thus, the sum of the IRs of a cation and an anion gives the same value as the sum of the CRs. Even in the field of material informatics being developed currently, IR and CR are often used as two of the most important descriptors of materials. If we assume rigid-sphere packing of ions with IR or CR, the bond length should be given as the sum of IR and CR for touching ions. Real crystals, however, would not be so simple. So Table I of Shannon (1976 ▸) lists IRs and CRs which are coordination-number (CN) dependent (Shannon, 1976 ▸). The idea of CN dependence of IR and CR may sound reasonable. However, there is still a limitation in applicability. In fact, Shannon gave a qualitative evaluation of IR and CR values in the last column of Table I (Shannon, 1976 ▸). We can examine how IR and CR work for our histograms of bond-length distributions.

Let us now explain the database and computational tools by which we obtained our bond-length distributions. We used the COD, which now contains more than 400 000 crystallographic information files (CIFs) (Hall *et al.*, 1991 ▸). One can freely download all the CIFs. The COD is a sophisticated database and is kept up to date thanks to the systems used to update and validate CIFs automatically (Vaitkus *et al.*, 2021 ▸). For our computational tool, we employ the program *ChemEnv* provided by Waroquiers *et al.* (2017 ▸, 2020 ▸) to specify local environments (Ganose & Jain, 2019 ▸; Zimmermann & Jain, 2020 ▸). *ChemEnv* is integrated into one of the most well known tool sets of material informatics, *pymatgen* (Ong *et al.*, 2013 ▸). By using *ChemEnv*, we can determine the local environments of cations from the coordination environments (CEs) in the crystal structures. The CE specifies how a cation is surrounded by nearest-neighbour (NN) anions. All program codes for the calculations with the results of this paper are freely available at our github site (https://github.com/tkotani/CEBond).

Illustrations of the CEs are listed in Fig. 1 of Waroquiers *et al.* (2017 ▸). When a CE is given, we can identify atomic bonds connecting the cation and the NN oxygens. Specifying a CE gives a more detailed description of how a cation is surrounded by the NN oxygens than a CN does. For example, one of the CEs, ‘T:4 Tetrahedral’, means that we have four NN oxygens (CN = 4) which surround the cation tetrahedrally. We can get the bond-length distribution with decomposition by the CEs. Our results are an extension of the automated analysis of CEs given by Waroquiers *et al.* (2017 ▸), where they show a pie chart with the percentage of CEs for each ion species. What we have developed here is a method of resolving their pie charts with an additional axis of bond length.

Bond-length analyses have been carried out by other groups. One of the latest pieces of work was done by Gagné and Hawthorne with a series of publications (Gagné & Hawthorne, 2016 ▸, 2020 ▸). Using the Inorganic Crystal Structure Database (ICSD; https://icsd.fiz-karlsruhe.de/index.xhtml), they analysed the bond-length distribution with decomposition by CN and ionic valence. Our results are essentially similar to theirs, although we have organized our codes and results for educational purposes.

In this paper, after explaining our analysis of the anion radii, we show the histograms of the bond-length distributions in oxides including alkali, alkali earth, and 3*d* and 4*d* transition metal elements. The results for fluorides and nitrides are given on our web site (https://github.com/tkotani/CEBond). In addition, we show the distributions of the bond lengths for Cu—O, Fe—O and V—O bonds with decomposition by ionic valence. Together with our results of histograms of bond-length distributions, we show the bond lengths calculated as the sum of IRs or CRs to see how the idea of IR and CR is justified.

## 2. Method

We first obtain a set of CIFs of oxides from the COD in two steps, a download step and a filtering step. In the download step, we download CIFs on a list returned for our query in SQL to the COD database (how to query the COD database is explained at https://wiki.crystallography.net/howtoquerycod/). In the query, we set the conditions for selecting the CIFs as follows:

(i) The temperature range is 270–370 K.

(ii) The pressure range is 0–1000 kPa.

(iii) To pick up oxides which include only oxygen as anions, we remove CIFs including elements N, F, P, S, Cl, As, Se, Br, Sb, Te, I, Bi, Po and At, in addition to H and C.

(Precisely speaking, we added further conditions as well; see *.sql at https://github.com/tkotani/CEBond.) The total number of downloaded CIFs of oxides was 16 313.

We then proceed to the filtering step, with the following checks:

(iv) Crystal structure check: correctly recognized by *pymatgen* or not (through *CifParser* in *pymatgen*).

(v) Occupancy check: each site is occupied by only one atomic species and the occupancy should be more than 90.0%.

(vi) Not in our skip list.

With this filtering process, the total number of 16 313 CIFs is reduced to 6992. In the filtering process, 610 CIFs caused errors on check (iv). In the skip list, we designated three CIFs which were broken or consumed too much time in the filtering process. The greatest number of CIFs were removed by check (v), the aim of which is to avoid ambiguity in our analysis of bond lengths. The resulting 6992 files are used in Section 3. Among these 6992 CIFs, 27 caused errors in the analysis using *ChemEnv*. The remaining 6965 files were used for our bond-length analysis presented in Sections 4 and 5. We have not tried to elucidate the reasons for the errors since the number 610 + 3 + 27 = 640 is small relative to the available 6965. We expect that our conclusions would not be altered if we resolved the errors. Details of the two steps are provided on our web site (https://github.com/tkotani/CEBond).

**Figure 1:**
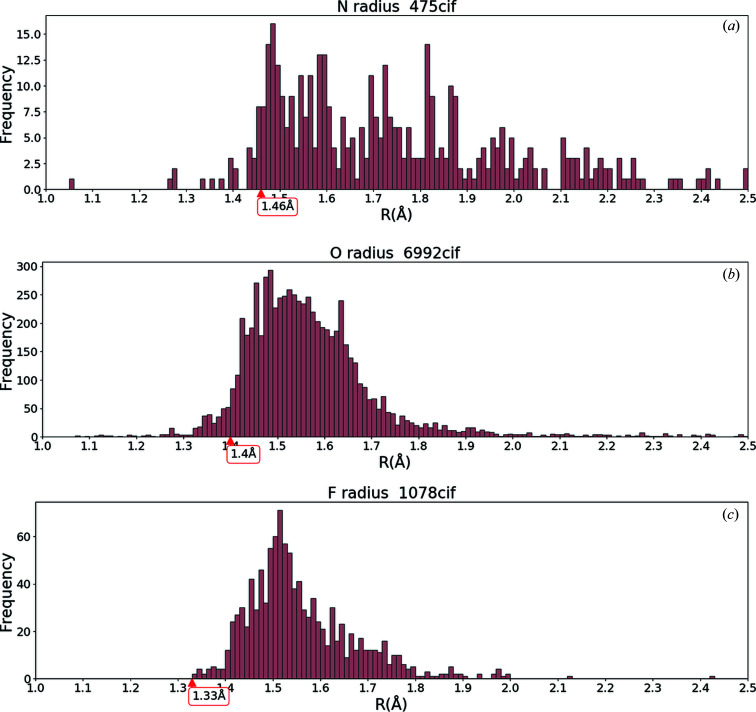
Frequency histograms of anion radius *R* calculated from equation (2), together with Shannon’s ionic radius (IR) (Shannon, 1976 ▸; table of data at http://abulafia.mt.ic.ac.uk/shannon/radius.php) for (*a*) nitrides, (*b*) oxides and (*c*) fluorides. See the text for further details.

The tool *ChemEnv* determines the CEs. Here we introduce a CE variable *C*_env_ which takes one of the CE names contained in the Ω_env_. That is, *C*_env_ ∈ Ω_env_, where Ω_env_ = {S:1 Single neighbour, L:2 Linear, A:2 Angular, TL:3 Trigonal plane, …}. The CE in Ω_env_ is shown in Fig. 1 of the report by Waroquiers *et al.* (2017 ▸). *C*_env_ not only specifies the atomic bonds connecting the central cation at **R** to the surrounding anions but also contains the shape of the polygons formed by the surrounding anions. With *ChemEnv*, we obtain the probability *P* ^**R**^(*C*_env_) of the CEs. For example, we have *P* ^**R**^(*C*_env_) = 80.0% for *C*_env_ = L:2 and *P* ^**R**^(*C*_env_) = 20.0% for *C*_env_ = A:2.

The algorithm to calculate the probability *P* ^**R**^(*C*_env_) is given in equation (1) of Waroquiers *et al.* (2017 ▸). For a given **R**, several *C*_env_ have non-zero *P* ^**R**^(*C*_env_), for which normalization is set as 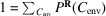 . Note that *C*_env_ with non-zero *P* ^**R**^(*C*_env_) may have different CNs. The atomic bonds are specified by (**R**, *C*_env_, *i*), where *i* is the index to distinguish atomic bonds starting at **R**; the range of *i* is dependent on *C*_env_.

From *P* ^**R**^(*C*_env_), we calculate the bond weight *w*(**R**, *C*_env_, *i*) as 

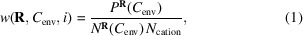

where *N* ^**R**^(*C*_env_) is the CN of *C*_env_, that is, the number of atomic bonds starting from the cation at **R**. *N*_cation_ is the number of cations in the primitive cell. Note that *w*(**R**, *C*_env_, *i*) is independent of *i* in equation (1) since we distribute an equal weight, 1/*N* ^**R**^(*C*_env_), for bonds in equation (1). The total weight of bonds is normalized to be unity for a CIF, 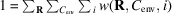 , where **R** runs over all cation sites in the primitive cell, *C*_env_ runs over all CEs in Ω_env_ and the summation 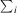 is equivalent to multiplying the factor *N* ^**R**^(*C*_env_).

For all the CIFs, the atomic bonds are specified by (*m*, **R**, *C*_env_, *i*), where *m* is the index of the CIF; the range of (**R**, *C*_env_) is dependent on *m*. We generate *w* ^*m*^(**R**, *C*_env_, *i*) for all the CIFs of oxides. Since *m* and **R** determine the atomic species *s*, we can obtain a set of atomic bonds Ω(*s*, *C*_env_): the atomic bond (*m*, **R**, *C*_env_, *i*) ∈ Ω(*s*, *C*_env_) is for a species *s* with *C*_env_. For bond lengths *l* for bonds in Ω(*s*, *C*_env_), we can construct a histogram 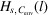 for a given *s* and *C*_env_ by accumulating the weight *w* ^*m*^(**R**, *C*_env_, *i*) along the histogram bins of *l*. We use 0.01 Å for the bin width of *l*. The sum 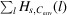 should match the percentages shown by the pie charts of Waroquiers *et al.* (2017 ▸).

## 3. Examination of anion radii

Before we show our main results of bond-length distributions, let us evaluate the radii of anions by the following method. If we assume a rigid-sphere model where the anions are closely packed and the cations are small enough to be negligible, we can calculate the anion radius *R* from the cell volume *V*_cell_ and the number of anion atoms per cell *N* from 

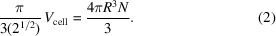

Here, π/[3(2^1/2^)] is the ratio of the closely packed rigid spheres to the cell volume. We can calculate *R* only from *V*_cell_ and *N* for a CIF.

Fig. 1 ▸ shows three frequency histograms of *R* calculated using equation (2). In Fig. 1 ▸(*b*) for oxides, we observe a sudden increase around *R* ≃ 1.4 Å, corresponding to the IR of O^2−^ for CN = 6. (We use a value at CN = 6 for simplicity. We neglect the CN dependence of the anions.) The sudden increase means the rigid-sphere packing model can be applicable for O^2−^ and thus we may use 1.4 Å as the radius of O^2−^. However, we need to caution that the number of CIFs whose *R* is below 1.4 Å is ∼300. This shows the limitation of the rigid-sphere packing model. In Fig. 1 ▸(*a*) for nitrides, a sudden increase occurs at 1.46 Å [= IR of N^3−^ (CN = 4)] as in the case of oxygen. In contrast, the IR of 1.33 Å for F^−^ (CN = 6) is located at the left-hand end of the tail of the histogram as shown in Fig. 1 ▸(*c*). In the case of fluorides, we see a narrower distribution than for oxides and nitrides. This can be interpreted as the fluorides favouring less covalency and the rigid-sphere packing model being more suitable than for oxides and nitrides.

## 4. Alkali and alkali earth elements

**Figure 2:**
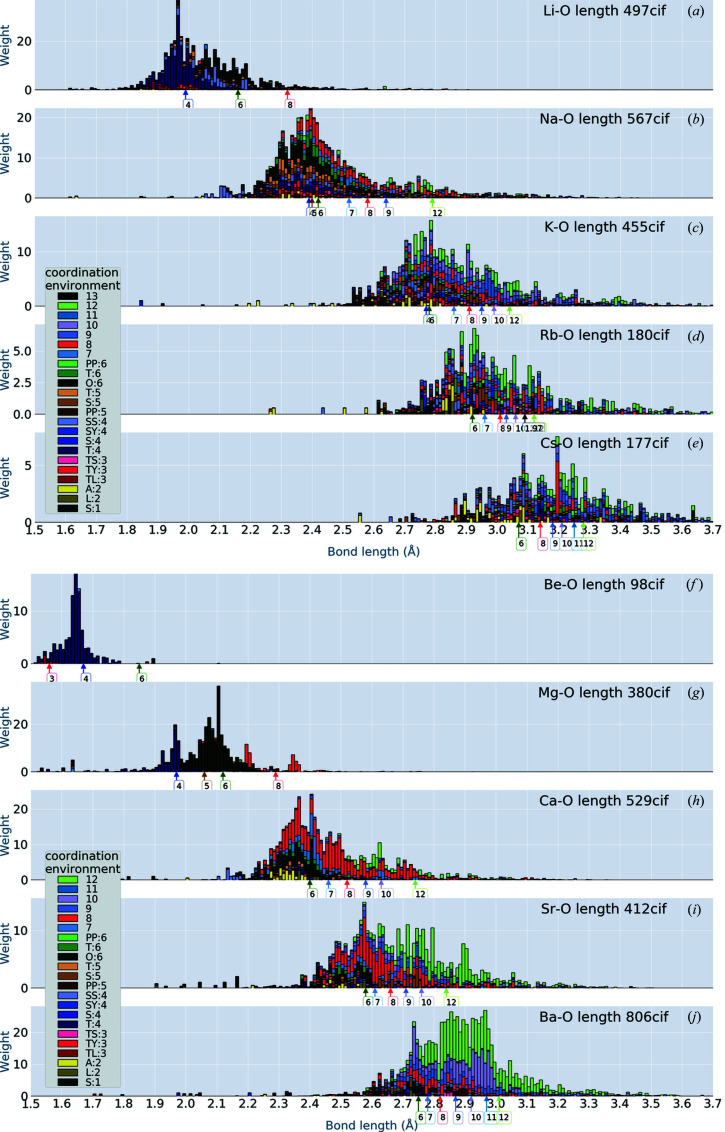
Bond-length distributions for alkali and alkali earth oxides. The coordination environments are coloured as shown in the insets. To distinguish similar colours, attention must be paid to the stacking order for some of the CEs: we use the stacking order in the insets. Arrows on the *x* axis show the bond lengths calculated from Shannon’s IRs (see the text for discussion). The weight accumulated to construct the distribution is given by equation (1).

Fig. 2 ▸ shows the bond-length distributions as histograms of 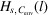 in oxides where the atomic species *s* is one of the alkali or alkali earth elements. The *x* axis is for the bond length *l*. We stack the components of *C*_env_ with different colours. Extreme outliers [*e.g.* the small weights below 2.3 Å in Fig. 2 ▸(*d*)] may or may not be real features because of the limitation of our method.

Our histograms show the chemical trends of the bond-length distribution quite well. We observe how the distributions change for different elements. In our histograms, we resolve CN < 7 into CEs (Waroquiers *et al.*, 2017 ▸) as

(i) S:1 Single neighbour,

(ii) L:2 Linear, A:2 Angular

(iii) TL:3 Trigonal plane, TY:3 Triangular non-coplanar, TS:3 T-shaped,

(iv) T:4 Tetrahedron, S:4 Square plane, SY:4 Square non-coplanar, SS:4 See-saw,

(v) PP:5 Pentagonal plane, S:5 Square pyramid, T:5 Trigonal bipyramid,

(vi) O:6 Octahedron, T:6 Trigonal prism, PP:6 Pentagonal pyramid,

where the numbers following the labels S:, L: *etc.* give the CN.

We observe some differences from the results of Gagné & Hawthorne (2016 ▸). For example, our distribution of Na—O bonds in Fig. 2 ▸(*b*) has a slightly larger weight on the longer bond lengths than the results shown by Fig. 1 of Gagné & Hawthorne (2016 ▸), although overall the distributions show reasonable agreement. Lengths for CN = 6 are distributed around 2.4 Å, in agreement with Fig. 5 in Gagné & Hawthorne (2016 ▸).

The bond lengths are widely scattered for a given CE. For example, we observe that CN = 8 for Ca—O [red histogram bars in Fig. 2 ▸(*h*)] ranges from 2.3 Å to 2.8 Å. We can observe differences among different atomic species: the histograms of Be and Mg are very different from those of Ca, Sr and Ba. For Li—O, we observe that relatively simple CEs dominate in the crystal structures. On the other hand, Na—O bonds take a variety of CEs with a relatively wider range of bond lengths. This result gives us the expectation that Na ions in oxides can be mobile by hopping sites with different CEs.

In Fig. 2 ▸, we have put arrows on the *x* axis to show the bond lengths which are given by the sum of the IRs of the cation and anion. For example, the arrow labelled ‘4’ in Fig. 2 ▸(*a*) points to 1.99 Å as the sum of IR = 0.59 Å for Li^+^ (CN = 4) and IR = 1.4 Å for O^2−^. (We use IR = 1.46, 1.4 and 1.33 Å for N, O and F, respectively; see Fig. 1 ▸.) As we might expect, the positions of these arrows are located almost at the centre of the bond distribution for the given CN.

## 5. Transition metals

**Figure 3:**
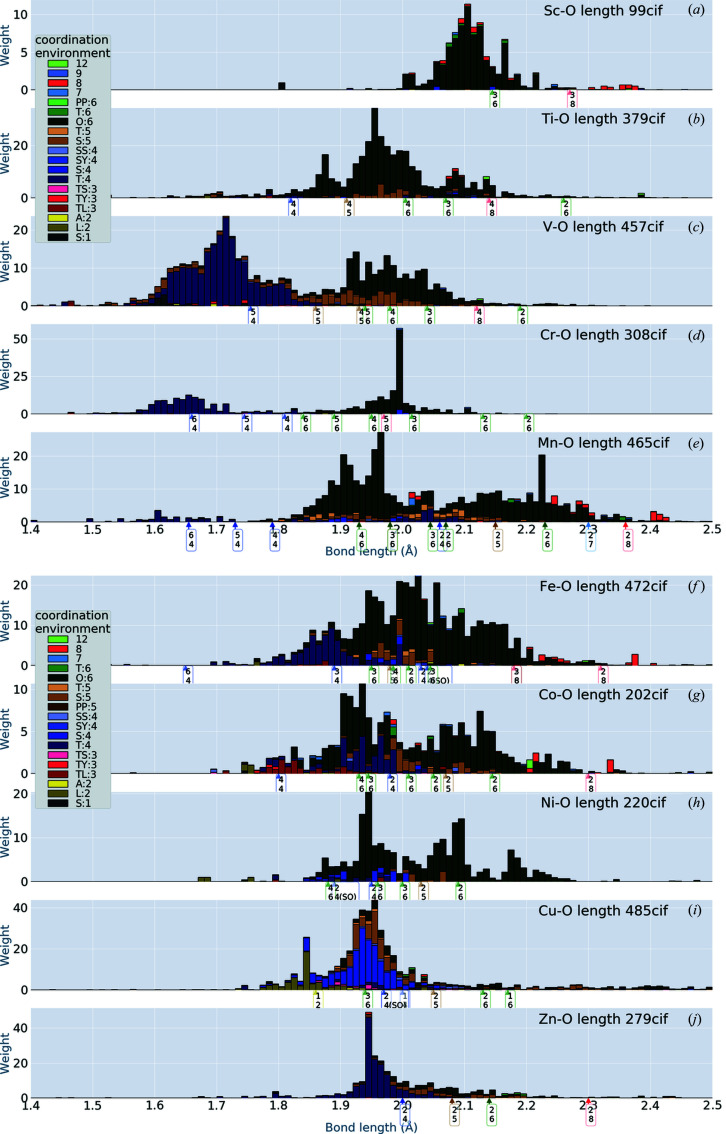
Bond-length distribution in ionic crystals including 3*d* transition metal elements. The coordination environments are coloured as shown in the insets. To distinguish similar colours, attention must be paid to the stacking order for some of the CEs: we use the stacking order in the insets. Arrows on the *x* axis show the bond lengths calculated from Shannon’s IRs (see the text for discussion). The arrows have labels with two numbers: upper numbers for ionic valence, lower for CN. The same tag can be at different bond lengths since spin dependence is not shown. The weight accumulated to construct the distribution is given by equation (1).

In Figs. 3 ▸ and 4 ▸ we show the distributions for transition metals. From our histograms, we can observe chemical trends that were not detected from the pie charts of Waroquiers *et al.* (2017 ▸). [Also compare our results with those of Gagné & Hawthorne (2020 ▸).]

Our histograms change as a function of atomic number in a systematic way. For example, the peaks of the T:4 distribution (dark-purple histogram bars) for V—O, Cr—O and Mn—O decrease systematically. Both Cr—O and V—O have peaks of CN = 4 and CN = 6 with similar heights, but the widths of the distributions are significantly narrower for Cr—O.

The histograms for Mn—O, Fe—O, Co—O and Ni—O also change systematically. Roughly speaking, the distribution of Mn—O has two broad peaks which come from O:6 (deep green). One is at 1.8–2.0 Å and the other at 2.1–2.3 Å. We observe two broad peaks in Co—O corresponding to those in Mn—O, but the corresponding peaks are unclear in Fe—O.

One of our findings is the similarity between V—O and Mo—O bonds, in spite of the fact that V and Mo belong to different columns of the periodic table. There is a similarity between Ti—O and Nb—O, and between Sc—O and Zr—O, as well. This may mean that Sc could be easily substituted with Zr in oxides, for example.

Arrows are shown with labels having two numbers: the upper number indicates the ionic valence and the lower the CN. [Corresponding to our analysis neglecting spin dependence, we put the same labels for arrows pointing to different lengths. For example, there are two arrows with the label ‘2 6’ for Co^2+^ (CN = 6) in Fig. 3 ▸(*g*). They point to 2.05 Å = 0.65 + 1.4 Å (low spin) and to 2.145 Å = 0.745 + 1.4 Å (high spin).] Let us look at Mn—O of Fig. 3 ▸(*e*), for example. The arrows for CN = 6 (ionic valence takes a value of 2, 3 or 4) are scattered over a broad range, corresponding to the bond-length distribution of O:6 (deep green).

**Figure 5:**
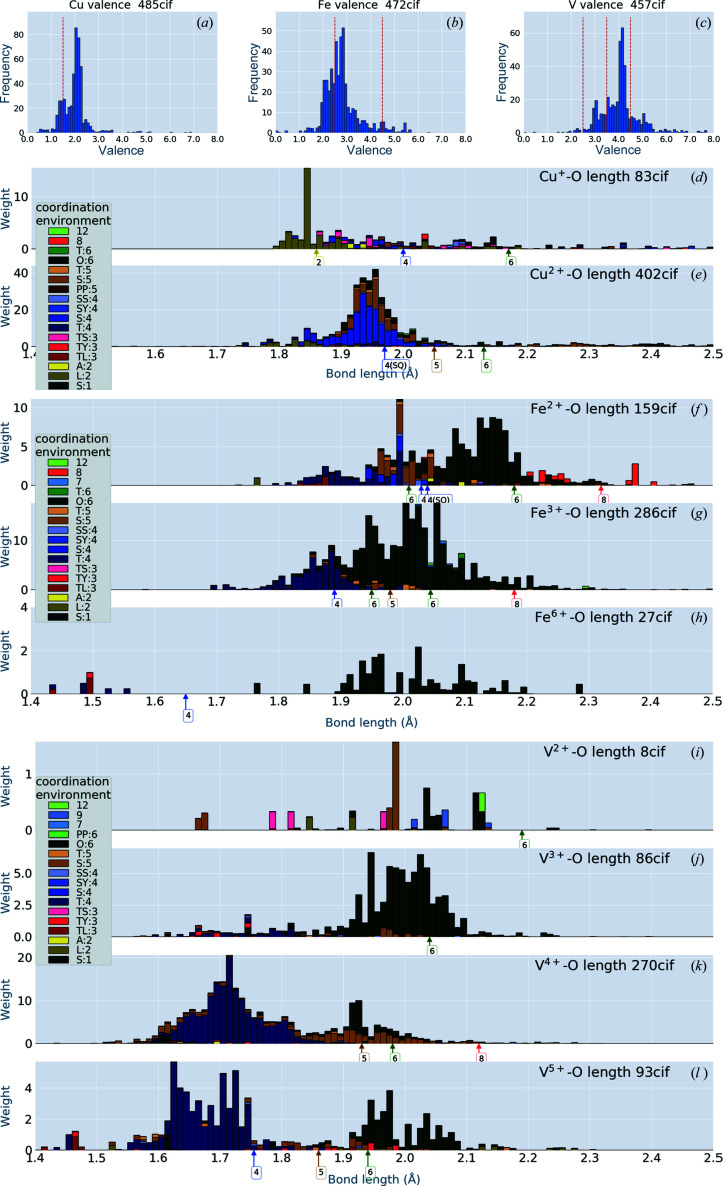
Bond-length distribution in ionic crystals including Cu, V and Fe, decomposed by ion valence. The topmost panels show histograms of valences calculated using the BVS method: vertical red dashed lines show the boundaries of valences to get the panels of Cu, V and Fe with valence-value decomposition. The sum of the histograms for each element reproduces the histograms in Fig. 3 ▸.

Fig. 5 ▸ shows the results of further decomposition of the V—O, Fe—O and Cu—O bonds of Fig. 3 ▸ into ion valences, where we have employed the bond-valence sum (BVS) method (Brown, 2009 ▸; Gagné & Hawthorne, 2015 ▸) to determine the ion valences. We have no better suggestion than the BVS to calculate ionic valences in compounds. In the BVS method, we determined the charge transfer between two atoms as a function of the length between the two atoms. Here we used a version of the function given by Gagné & Hawthorne (2015 ▸). The function is dependent on the two atomic species connected by the bond. Using the sum of charge transfer between the two atoms, we can calculate the ionic valence of the atom. Apparently, the sum is not of integer value, as shown in the top panel of Fig. 5 ▸. We have drawn vertical broken lines at valence = 1.5 to show the boundary value for distinguishing Cu^+^ and Cu^2+^. The same lines are shown for V and Fe. With this definition of the valences, we have obtained valence-decomposed histograms for Cu, Fe and V. According to the formula for BVS given by Gagné & Hawthorne (2015 ▸), a smaller valence favours a smaller CN or a longer bond length. In the case of Cu, Figs. 5 ▸(*d*) and 5 ▸(*e*) show that CN = 2 (smaller CN) is mostly selected for Cu^+^. Comparison of Figs. 5 ▸(*f*) and 5 ▸(*g*) shows that shorter bond lengths are favoured for larger valences.

Note that the valences calculated from the BVS are considered to be parameters to measure how cations are surrounded. A longer bond length and a lower number of neighbours give the same effect on the BVS. That is, we get the same BVS results for a cation when we move some neighbouring oxygens away from the cation and simultaneously move some other neighbouring oxygens closer: the idea of BVS is that such movements do not change the environment of the cation in view of the valences.

## 6. Summary

We have shown how to get histograms of the bond-length distributions in ionic crystals whose structural data are available in the COD. The histogram bars are decomposed in terms of CEs. We can observe the chemical trends of the bond-length distributions. Our results show the scattering of bond lengths, which is not accessible using the idea of ionic radius. We have found some interesting features. For example, Mo—O and V—O show similar types of distribution, although Mo and V are in different columns of the periodic table. The difference between Li—O and Na—O may suggest that Na is better than Li for ionic transport in oxides. Similarity between Sc—O and Zr—O indicates that Sc can be substituted with Zr in oxides. Our results will offer better understanding of ionic crystal structures, which will facilitate the development of new materials.

## Figures and Tables

**Figure 4:**
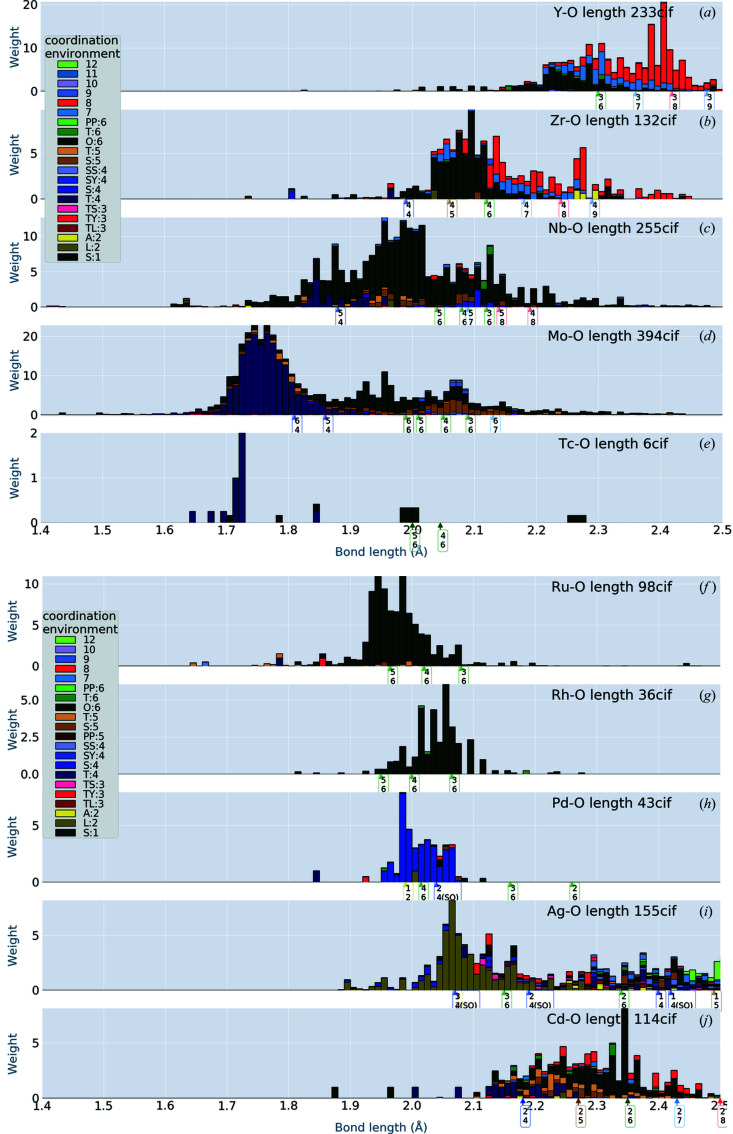
Bond-length distribution in crystals including 4*d* transition metal elements. The coordination environments are coloured as shown in the insets. To distinguish similar colours, attention must be paid to the stacking order for some of the CEs: we use the stacking order in the insets. Arrows on the *x* axis show the bond lengths calculated from Shannon’s IRs (see the text for discussion). The arrows have labels with two numbers: upper numbers for ionic valence, lower for CN. The same tag can be at different bond lengths since spin dependence is not shown. The weight accumulated to construct the distribution is given by equation (1).
